# NRF2 Regulation Processes as a Source of Potential Drug Targets against Neurodegenerative Diseases

**DOI:** 10.3390/biom10060904

**Published:** 2020-06-14

**Authors:** Ángel Cores, Marta Piquero, Mercedes Villacampa, Rafael León, J. Carlos Menéndez

**Affiliations:** 1 Unidad de Química Orgánica y Farmacéutica, Departamento de Química en Ciencias Farmacéuticas, Facultad de Farmacia, Universidad Complutense, 28040 Madrid, Spain; acores@ucm.es (Á.C.); martapiquero@ucm.es (M.P.); mvsanz@ucm.es (M.V.); 2 Instituto Teófilo Hernando y Departamento de Farmacología y Terapéutica, Facultad de Medicina, Universidad Autónoma de Madrid, 28029 Madrid, Spain; rafael.leon@inv.uam.es; 3 Instituto de Investigación Sanitaria, Servicio de Farmacología Clínica, Hospital Universitario de la Princesa, 28006 Madrid, Spain

**Keywords:** NRF2–ARE pathway, neurodegenerative diseases, oxidative stress, KEAP1, NRF2 regulation

## Abstract

NRF2 acts by controlling gene expression, being the master regulator of the Phase II antioxidant response, and also being key to the control of neuroinflammation. NRF2 activity is regulated at several levels, including protein degradation by the proteasome, transcription, and post-transcription. The purpose of this review is to offer a concise and critical overview of the main mechanisms of NRF2 regulation and their actual or potential use as targets for the treatment of neurodegenerative diseases.

## 1. Neurodegenerative Diseases and Oxidative Stress as Common Pathological Pathway

Over the past decades, life expectancy has been continuously increasing with the subsequent increase in the prevalence of aging-related chronic diseases, such as Alzheimer’s (AD), Parkinson’s (PD) and Huntington’s (HD) diseases, Friedrich’s ataxia (FRDA) amyotrophic lateral sclerosis (ALS), multiple sclerosis (MS) or stroke, among other types of neurodegenerative maladies [1]. In contrast to the traditional approach of “one disease–one mechanism–one medicine”, chronic diseases have a multifactorial etiology, requiring much more precise knowledge of the mechanisms involved in disease development [1].

The development of human disease networks has underscored the fact that different diseases (NDDs) may share common molecular mechanisms [2]. Thus, neurodegenerative diseases are characterized by a loss of homeostasis involving mitochondrial disfunction, chronic inflammation, metabolic and proteostasis unbalances associated with an increase of oxidative stress, and the pathological formation of reactive oxygen species (ROS) [1].

In this context, there is large body of evidence that points to nuclear factor (erythroid-derived 2)–like 2 (NRF2) as an attractive, druggable target that is involved in the regulation of multiple cytoprotective responses [1]. In this review, we will present the different mechanisms involved in NRF2 regulation to provide new strategies for drug development against neurodegenerative diseases (NDDs). The regulation of activity of NRF2 is complex, including control of its proteasomal degradation and the transcriptional and post-transcriptional levels [3].

## 2. The NRF2–ARE Pathway

After the discovery, in 1994, of the NRF2 transcription factor [4], its involvement in the regulation of the expression of about 250 genes containing an enhancer sequence called antioxidant response element (ARE) was shown [5]. These genes encode several enzymes involved in cellular protection against pro-oxidants, electrophiles or inflammatory agents, biotransformation reactions of xenobiotics, maintenance of mitochondrial function, protein homeostasis, and antioxidative metabolism [6].

**Figure 1:**
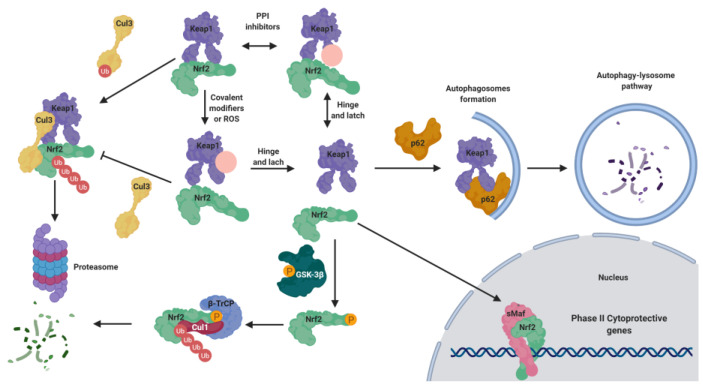
Negative regulation of nuclear factor (erythroid-derived 2)–like 2 (NRF2) under normal conditions and activation under pathological conditions. Under normal conditions, NRF2 is bound to Kelch-like ECH-associated protein 1 (KEAP1), maintaining it in the cytosol. KEAP1 acts as adaptor of the Cullin 3 (CuI3) ubiquitin ligase that ubiquitinates NRF2, leading to its degradation by proteasome 26S. Under pathological conditions, in the presence of reactive oxygen species (ROS) or covalent modifiers, the KEAP1 structure is modified, liberating NRF2 in the cytosol; thereafter, it translocates to the nucleus where it forms heterodimers with small masculoaponeurotic fibrosarcoma (sMaf) proteins to bind the antioxidant response element (ARE) sequences, promoting the expression of phase II genes. This mechanism is known as the “hinge and latch” NRF2 activation mechanism. Moreover, NRF2 is tightly regulated by additional mechanisms. It can be over-activated by the action of p62, since it can induce KEAP1 degradation by the autophagy lysosome pathway. In the opposite, under pathological conditions, in which the kinase glycogen synthase kinase-3β (GSK-3β) is over-active, it can directly phosphorylate NRF2, facilitating its interaction with the Cul1 ligase adaptor β-transducing repeat-containing protein (β-TrCp) to induce its degradation.

The maintenance of NRF2 levels requires a suitable balance between its synthesis and its proteasomal degradation [7,8]. As summarized in Figure 1, under physiological conditions NRF2 is normally located in the cytosol [7]. KEAP1 (Kelch-like ECH-associated protein 1), the NRF2 main negative regulatory system [7], is able to maintain NRF2 in the cytoplasm to generate a complex with the Cullin 3 (Cul3)/Rbx1-based E3-ubiquitin ligase, inducing NRF2 ubiquitination and the subsequent proteasomal degradation [8]. Thus, NRF2 is constitutively synthesized and rapidly degraded by the proteasome under unstressed conditions [8]. However, in response to oxidative stress, the cysteine residues present in the “sensor” region of KEAP1 are oxidized, leading to a conformational change that releases NRF2 from KEAP1. Alternatively, these cysteine residues can be activated by reaction with electrophiles [9]. Once NRF2 is liberated from KEAP1, it translocates into the nucleus, where it forms heterodimers with different coactivators, such as the sMaf (small masculoaponeurotic fibrosarcoma) protein [1,9], and these heterodimers promote the transcription of ARE. Due to its cytoprotective effects, NRF2 activation stands out as a promising goal to fight neurodegeneration processes, which involve mitochondrial disfunction, chronic inflammation, and increased oxidative stress levels [10].

## 3. Structure of NRF2

**Figure 2:**
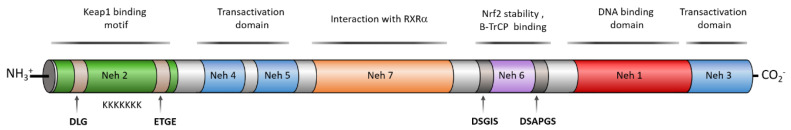
Human NRF2 domain structure and the activity associated to each domain. NRF2 is composed by seven Neh domains with different interaction patterns and specific activities. Neh1 presents a bZip region involved in dimerization with sMaf proteins to bind ARE regions and other transcription factors such as c-JUN, Sp-1, and JDP2 (Jun dimerization protein 2). Neh1 is also an acetylation-sensitive domain, contains an NES sequence to be exported by Crm1 and it is the target of phosphorylation by AMPK (AMP-activated protein kinase). Neh2 is a domain for degradation (degron domain) that presents the binding domain of KEAP1 and other ubiquitin ligases. KEAP1 targets the DLG and ETGE motifs each of them with different affinity. Neh 2 contains a α-helix composed by 7 Lys residues that are the targets for ubiquitination, an NLS sequence, and a serine residue (S40), that is a phosphorylation target of PKCδ, and both motifs are related to NRF2 nuclear translocation. The Neh3 domain contains another NLS sequence and two Lys residues that are targets for acetylation related to nuclear translocation. Neh4 and Neh5 are transactivation domains involucrate in the binding of CBP, P300, Hrd1, and RAC3 to NRF2. Neh6 contains a second degron domain targeted by β-transducing repeat-containing protein (β-TrCP) by interaction with the DSGIS motif, after being phosphorylated by GSK-3β and the DSAPGS motif. Finally, Neh7 is a repressor domain targeted by RXRα.

NRF2 belongs to the basic-region leucine zipper (bZIP) transcription factors, and more specifically to the cap ‘‘n’’ collar (CNC) subfamily [11]. It is formed by seven NRF2–ECH homology domains known as Neh1–7, each with a specific function (Figure 2) [12]. The Neh1 domain contains the CNC–bZIP region that allows NRF2 to recognize DNA, promoting its heterodimerization with partners such as the sMaf protein [13]. The high degree of conservation found in the amino acid sequence of this region across a wide range of species [14] highlights the importance of NRF2 transcriptional activity. Neh2 is responsible for the interaction with KEAP1, the main NRF2 negative regulator [15]. This interaction takes place through the degron motifs present in this domain, namely DLG (low affinity) and ETGE (high affinity) [15]. Moreover, the presence of seven lysine residues, also included in the Neh2 region, promotes NRF2 proteasomal degradation after its ubiquitylation [9]. The C-terminal Neh3, Neh4, and Neh5 are transactivation domains that bind to various components of the transcriptional machinery, which promotes the transcription of NRF2 target genes [16,17]. Neh6 is a serine-rich region containing two conserved peptide motifs, DSGIS and DSAPGS [18]. Glycogen synthase kinase-3β (GSK-3β) is able to phosphorylate the DSGIS sequence, thereby increasing the binding efficiency of β-transducing repeat-containing protein (β-TrCP) to Neh6 and promoting NRF2 proteasomal degradation [18]. Finally, Neh7 is responsible for the binding with RXRα (Retinoic acid receptor-alpha) and disrupts the binding of CBP ([CREB (cAMP-response-element-binding protein)-binding protein]) to the Neh4 and Neh5 domains, inhibiting the transcription of the ARE sequences [19].

The classical NRF2 target genes encode enzymes distributed in several organelles and subcellular compartments [20]. These enzymes participate in metabolic reactions that scavenge ROS and neutralize electrophiles such as superoxide dismutase (SOD), glutathione peroxidase, catalase, glutathione reductase (GR), glutamate cysteine ligase (GCL), or NAD(P)H/quinone oxidoreductase 1 (NQO1) [20]. NRF2 also plays a key role in the induction of genes involved in drug metabolism and distribution, including genes encoding non-cytochrome P450 phase-I and phase-II drug metabolism enzymes [21]. Furthermore, it promotes the degradation of the heme group by inducing heme oxygenase-1 (HO-1), and some of the induced enzymes are involved in the generation of antioxidant small molecules such as glutathione synthase, which is essential for the biosynthesis of glutathione (GSH), the most abundant antioxidant and electrophile-neutralizing molecule inside the cells [22]. NRF2 also regulates proteins related to key processes such as autophagy [23], promotes the preservation of mitochondrial functions [24], and inhibits the expression of proinflammatory cytokines [25].

## 4. Neurodegenerative Diseases Hallmarks and Their Crosstalk with NRF2

### 4.1. Oxidative Stress

While moderate concentrations of ROS play physiological roles as second messengers [26], an imbalance of ROS concentration leads to oxidative stress and is related with the onset of some pathological processes. Oxidative stress is one of the major hallmarks in neurodegeneration and arises from the combination of ROS hyperproduction with the impairment of antioxidant defenses [27]. This imbalance causes molecular and organelle damage, leading into neuronal death if severe. ROS species, arising from the oxidative metabolism of O_2_, react with unsaturated fatty acids such as linoleic acid and arachidonic acid [27]. The resulting highly reactive peroxy radicals initiate a chain reaction with nearby fatty acids [28]. Some of these molecules arising from the ROS-promoted chain reactions are characteristic of specific NDDs, such as 4-hydroxy-2-nonenal (HNE) in AD and PD [28,29], acrolein and F_2_-isoprostanes in AD [30], and malondialdehyde in PD. DNA is also susceptible to suffer oxidative damage, as shown by the increased levels of 8-hydroxyguanine and 8-hydroxy-2-deoxyguanosine in the brains of PD patients [31]. Acrolein and HNE induce toxicity by crosslinking to cysteine, lysine, and histidine residues through Michael additions [32]. The formation of these adducts damages enzymes and receptors, and it induces DNA mutations, leading to malfunction of a variety of biochemical pathways [32]. Moreover, ROS induce structural alterations in proteins that lead to their misfolding and aggregation [33]. These protein alterations also cause the malfunction of complexes such as voltage-dependent calcium channels (VDCC) and N-methyl-D-aspartate (NMDA) receptors, contributing to intracellular calcium overload and excitotoxicity [33]. Furthermore, oxidative stress activates redox-sensitive pathways, leading to sustained M_1_ microglial activation [34].

Cells have developed a number of detoxifying mechanisms to maintain cellular redox homeostasis by inducing the transcription of phase I (cytochrome P450s) and phase II (detoxifying and antioxidant proteins) enzymes [34]. As described above, NRF2 is a master regulator in cellular redox homeostasis, and the activation of this pathway is an effective mechanism against oxidative or electrophilic stress [35].

### 4.2. Neuroinflammation

#### 4.2.1. NRF2 and Neuroinflammation: General Aspects

Inflammation plays a critical role in protection against tumors and infections and is essential for the reparation of tissues; however, chronical inflammation can be detrimental and is a common feature in neurodegeneration processes [36,37].

Neuroinflammation processes are mediated, mainly, by microglial cells switching from the resting state M_0_ to activated states (M_1_ or M_2_) by two different activation pathways [38]. The proinflammatory M_1_ phenotype releases proinflammatory cytokines such as IL-1β, IL-6 and the tumor necrosis factor-α (TNF-α) and increases ROS formation, leading to tissue damage [38]. An alternative activation route promotes the neuroprotective M_2_ state, which is involved in tissue regeneration processes and is characterized by the production of anti-inflammatory cytokines (IL-4, IL-10, and IL-13) [38]. The imbalance between M_1_/M_2_ phenotypes is being extensively studied to understand the complex role of microglia as a neuroprotective or neurodegenerative factor [39].

NRF2 activation exerts anti-inflammatory properties by reducing the amounts of several proinflammatory cytokines such as TNF-α, IL-1β (interleukin 1β), IL-6, and the expression of nitric oxide synthase (iNOS) in microglia and astrocytes [10,39]. It has been shown that the induction of the NRF2 pathway in the hippocampus and cortex of AD (APP/PSEN1) mice model [40] reduces microgliosis, astrogliosis, and the secretion of proinflammatory cytokines TNF-α and IL-17A. Most importantly, long-term memory decline of AD mice was significantly attenuated [40]. Furthermore, several human studies have demonstrated the cytoprotective properties toward Aβ peptide-induced toxicity achieved by NRF2 activation and related to its anti-inflammatory effects due to a shifting of the microglia state from the M1 to an M2 phenotype [41]. NRF2 activation has been shown to be useful in neuroinflammation attenuation, neuronal survival, and repressing the deleterious effects of activated microglia in ALS (amyotrophic lateral sclerosis) mouse models [42]. Similarly, pharmacologically stimulated NRF2 activation repressed inflammatory responses in mouse microglia and astrocytes, the principal cellular mediators of neuroinflammation, and in blood monocytes from HD patients [43]. NRF2 also interferes with two of the main proinflammatory pathways, as detailed below.

#### 4.2.2. Nrf2 and Toll-Like Receptor Signaling

**Figure 3:**
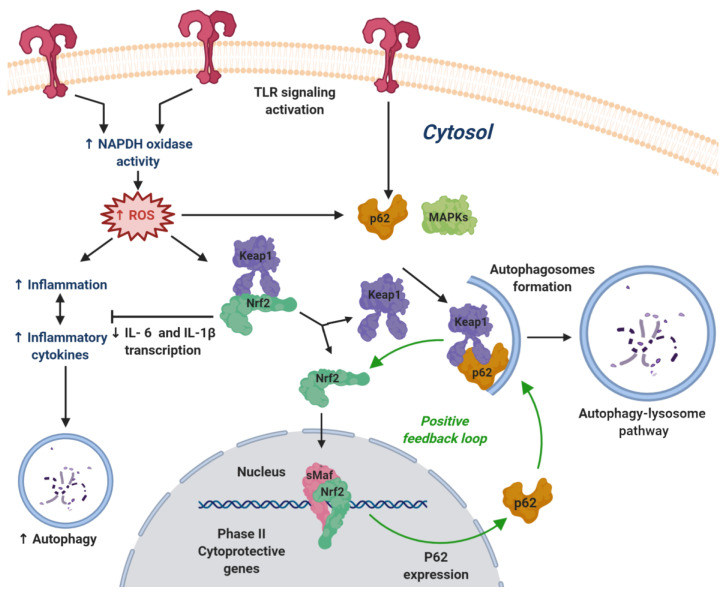
Crosstalk between the Toll-like receptor (TLR) and nuclear factor (erythroid-derived 2)-like 2 (NRF2) pathways. TLR signalling initiation induces the activation of NOX (NADPH oxidase) enzymes to produce high quantities of ROS that, in turn, react with sensor Cys residues present in KEAP1 inducing NRF2 liberation and nuclear translocation to activate the phase II antioxidant response. NRF2-regulated genes help to stop the proinflammatory response, reducing the production of proinflammatory cytokines and decreasing the formation of free radicals. TLR activation and ROS production increase the expression of p62 and the activity of mitogen-activated protein kinase (MAPKs). The p62 protein targets KEAP1 to induce its degradation via autophagy, favoring the activation of the NRF2–ARE pathway. In turn, NRF2 increases the expression of different proteins involved in the autophagic flux, including p62, generating a positive feedback loop to potentiate the anti-oxidant phase II response to resolve the proinflammatory signaling induced by TLR activation.

Toll-like receptors (TLRs) initiate a proinflammatory immune response through the activation of transcription factors such as the nuclear factor-κB (NF-κB) and others, which are associated to the synthesis of proinflammatory molecules (Figure 3). Several types of immune cells involved in TLR signaling are present in the brain, and they have an important role in the development of pathological neuroinflammation responses in a variety of NDDs, including AD, PD, and autoimmune processes such as MS [44].

The existence of crosstalk between TLR signaling and the NRF2 pathway is well documented, and the concerted action of both pathways allows a fine-tuned control of the anti-inflammatory response. As summarized in Figure 3, the connections between TLR signaling and the NRF2 pathway arise mainly from ROS generation associated to NADPH oxidase or from the induction of p62 or mitogen-activated protein kinase (MAPK) kinases. The overall process regulates inflammation through the modulation of cytokines via autophagy, by inhibiting the transcription of pro-inflammatory cytokines and by NRF2-mediated expression of anti-inflammatory molecules [45].

**Figure 4:**
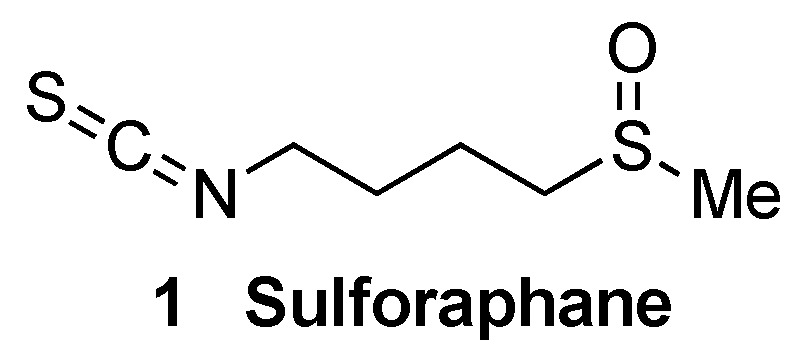
Structure of sulforaphane, a multitarget compound that is able to inhibit TLR3-mediated NF-κB signaling and to induce NRF2 by covalently binding to KEAP1.

It is interesting to note that some compounds that induce NRF2 are also able to modulate TLR activity, leading to an overall improved anti-inflammatory profile. Sulforaphane (compound **1**, Figure 4) is a good example, being able to inhibit TLR3-mediated NF-κB signaling and to induce NRF2 by covalently binding to KEAP1 [46], as will be further discussed in Section 5.1.2. Polyphenols provide another example of such multitarget behavior [47].

#### 4.2.3. NRF2 and NFκB

**Figure 5:**
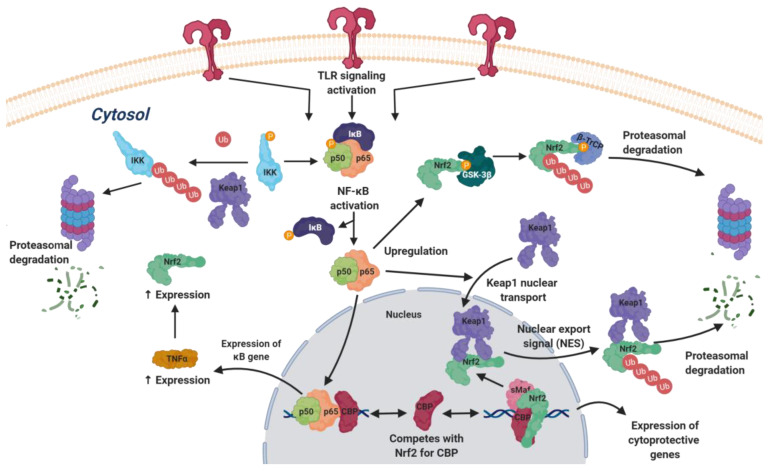
Crosstalk between the nuclear factor-κB (NF-κB) and NRF2 pathways. Upon TLR activation, NF-κB is liberated from its natural repressor, IκB, by phosphorylation promoted by the IKK (IκB kinase) complex. Liberated NF-κB dimer rapidly translocates into the nucleus where it binds to CREB binding protein (CBP), competing with NRF2, and it initiates the expression of proinflammatory genes including tumor necrosis factor-α (TNF-α) citokine. NF-κB drives a negative regulation over NRF2 through its monomer p65 by different pathways. p65 promotes the nuclear importation of KEAP1, where it binds to NRF2, concluding the expression of phase II-related genes. Then, the KEAP1–NRF2 complex is exported to the cytosol and coupled to the Cul3 ubiquitin ligase that induces the proteasomal degradation of NRF2. Additionally, GSK-3β directly phosphorylates NRF2, coding its degradation though interaction with β-TrCP. β-TrCP is also able to induce the degradation of IκB (inhibitor of nuclear factor kappa B), increasing the activity of NF-κB and thus limiting NRF2 activation. On the other hand, KEAP1 stabilizes IκB by inducing the degradation of IKK via proteasomal degradation. Thus, it helps to close the crosstalk between NF-κB and NRF2, creating a fine-tuned loop between inflammation and the antioxidant response.

The nuclear factor-κB family comprises several proteins that are activated downstream of TLR signaling and lead to an inflammatory response by the expression of proinflammatory cytokines such as TNFα and several interleukins. Its connection with NRF2 is very complex, and its main features are summarized in Figure 5 [48].

NF-κB exerts a negative effect on NRF2-driven gene expression through p65, which acts on the NRF2 pathway by several mechanisms [48]. On one hand, p65 competes with NRF2 for CREB binding protein (CBP), a transcriptional co-activator that acts by histone acetylation [49]; on the other hand, p65 promotes the transport of KEAP1 to the nucleus, where it binds to NRF2 and removes it from the nucleus via the nuclear export signal (NES) [49]. On the other hand, p65 is able to increase the expression of the NRF2 gene via increased TNFα levels.

### 4.3. Proteostasis

The misfolding of specific proteins and their subsequent aggregation in a toxic form is the most common feature of NDDs. These aberrant proteins arise from cell stress, mutations, or protein production mistakes [50]. The proteostatic system regulates the synthesis, proper folding, degradation, and clearance of proteins; however, during aging, the accumulation of misfolded proteins collapses the proteostatic system and increases the amount of ubiquitinated inclusion bodies (IBs) [51]. Depending on the disease, different protein aggregates are found such as α-synuclein (α-syn) in PD, β-amyloid (Aβ) plaques and hyper-phosphorylated tau neurofibrillary tangles (NFTs) in AD, huntingtin (Htt) in HD, superoxide dismutase 1 (SOD1), and TAR DNA binding protein 43 (TDP-43) in ALS and scrapie prion protein (PrP^Sc^) in spongiform encephalopathies [52]. Strong links have been found between the formation of misfolded protein deposits and adaptive immunity [53].

An interesting crosstalk exists between NRF2 and the protein degradation pathways [54]. The NRF2-mediated transcription of the components of these systems [23,55] enhances the degradation of misfolded proteins, while on the other hand, NRF2 is downregulated by its proteasomal degradation and also via autophagy [54]. Several in vitro and in vivo models have proved the protective role of NRF2-mediated pharmacological activation of protein clearance, which increases the degradation of phosphorylated and insoluble Tau through autophagy [56, 57]. Furthermore, sulforaphane (**1**, Figure 4), an NRF2 inducer, enhanced proteasomal degradation of mHtt (mutant huntingtin protein) through NRF2 induction [23]. Finally, it has been demonstrated that the neuroprotective effect of dimethyl fumarate, an approved drug for the treatment of MS that will be further discussed in Section 5.1.2, is in part caused by autophagy activation in an α-synucleinopathy model of PD [58].

## 5. NRF2 Activation as a Useful Approach to Neurodegenerative Disease Therapy

NRF2 deficiency has been found in several NDDs; for instance, the hippocampal neurons of AD patients showed a dramatic decrease in nuclear NRF2 [59], while animal models of PD, such as NRF2-knockout mice, showed a specific loss of dopaminergic neurons [60] and post-mortem studies of patients with ALS showed an increased KEAP1 mRNA in the motor cortex [61], leading to a decline in NRF2 activity. Other studies showed that tau- and amyloid-injured neurons contain increased levels of NRF2 and its target protein sequestosome 1 (SQSTM1/p62), probably as a clearance mechanism to release these toxic proteins through autophagy [23,62]. In consonance with these results, the levels of HO-1, NQO1, GCLM, and SQSTM1 levels are increased in AD and PD brains [62,63]. In this context, it is not surprising that NRF2 activation has been demonstrated to extend survival in AD [64], PD [65], and ALS [42] animal models.

The low levels in the expression or activity of NRF2 in neurodegeneration models together with the decrease of the neurodegenerative process upon NRF2 induction indicate that the NRF2–ARE pathway is a promising target for NDDs [35,66]. Furthermore, this pathway regulates multiple pathological processes implicated in neurodegeneration processes such as oxidative stress, neuroinflammation, and aberrant proteostasis, by targeting NRF2, several pathways can be simultaneously modulated [23,25,35,54].

### 5.1. KEAP1-Dependent Regulation

NRF2 regulation is accomplished by several cellular factors controlling its stability and nuclear translocation, but among them, the previously mentioned KEAP1 protein is the most important [9].

#### 5.1.1. KEAP1 as an Oxidative Stress Sensor

**Figure 6:**
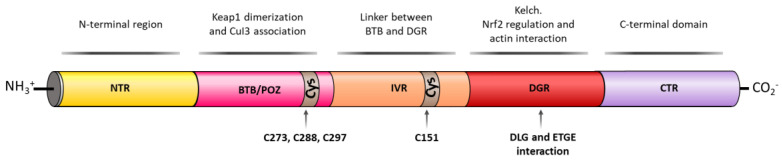
KEAP1 structural domains and their related activity. KEAP1 contains an N-terminal region (NTR), a Tramtrack and Bric-á-Brac (BTB) domain that enables KEAP1 homodimerization and responsible for Cul3 E3 ligase binding. The BTB domain also contains three important Cys residues (Cys^273^, Cys^288^, and Cys^297^) able to react with free radicals and/or electrophiles inducing a conformational change in KEAP1 that liberates NRF2 in the cytosol. The next domain is an intervening region (IVR) that also contains an important Cys residue, Cys^151^, which is able to react with free radicals and or electrophiles and an NES motif that regulates the cytoplasmatic location of KEAP1. The double glycine repeat (DGR) domain contains six kelch repeats that contain the binding sites of NRF2, p62, and related E/STGE proteins. Finally, KEAP1 contains the C-terminal domain (CTR).

KEAP1 is a Zn metalloprotein with 625 amino acid residues and it has a branched stem dimeric structure, formed by 5 domains [67]. The N-terminal region (NTR), the BTB/POZ (Bric-a-brac, tramtrac, broad-complex/proxvirus zinc fingers) domain, implicated in KEAP1 homodimerization and Cul3 association, the intervening region (IVR), which acts as a linker between BTB and DGR domains, the double glycine repeat (DGR) domain, important for NRF2 regulation and actin interaction and the C-terminal domain (CTR) [67] (Figure 6).

KEAP1 is a cysteine-rich protein, being redox-active and reactive to the local environment [68]. The majority of KEAP1 cysteine residues are surrounded by basic amino acids that increase their reactivity by lowering their pKa values [69]. Thus, the 27 cysteine residues of KEAP1 are proposed to be the main sites through which the protein is able to sense electrophilic or oxidative stress [70]. Even though cysteines are distributed along the whole KEAP1 sequence, it has been proved that the C^273^, C^288^, and C^297^ residues in the BTB/POZ domain [71] and C^151^ in the IVR domain [72] are more reactive and serve as redox sensors for electrophiles, reactive oxygen species (ROS), and metals such as Cd^2+^, As^3+^, and Se^4+^ [73]. The chemical modification of their sulfhydryl groups decreases NRF2 ubiquitination, causes KEAP1–NRF2 dissociation, and promotes the nuclear translocation of NRF2 [74].

The ‘hinge and latch’ model [75] is the most widely accepted mechanism for KEAP1-dependent regulation. In this model, two different binding sites of NRF2 in the Neh2 domain interact with different affinities with a single overlapping site in the DGR domain of KEAP1, with the ETGE motif presenting higher affinity than DLG [76]. Under homeostatic conditions, the β-hairpin of the ETGE motif (high affinity) interacts with one of the KEAP1 subunits in a first step, which is called the open conformation [76,77]. This open conformation represents the “hinge”, in which NRF2 can move in space relatively freely. In a second step, the β-hairpin of the DLG motif (low affinity) interacts similarly with a second protomer of KEAP1 in the so-called closed conformation, representing the “latch” [76,77]. This closed conformation restricts the ability of NRF2 to move and enables an optimal positioning of a Lys-rich α-helix present in the Neh2 region of NRF2; this helix is the target for ubiquitin conjugation, consequently promoting NRF2 for proteasomal degradation [77].

Under oxidative stress conditions, NRF2 inducers oxidize the Cys residues present in KEAP1, promoting a conformational change [77]. This structural change in KEAP1 causes an improper spatial disposition of the target lysines, and thus NRF2 is no longer ubiquitinated nor degraded [78]. This leads to the saturation of KEAP1, and consequently any newly synthesized NRF2 can evade repression and directly accumulate into the nucleus, promoting its target gene expression [79].

#### 5.1.2. KEAP1 Covalent Modifiers

While NRF2 can be physiologically activated by an increase of oxidative stress, it can also be exogenously induced by chemical agents [76]. The majority of known NRF2 inducers are electrophiles that modify the cysteine residues present in the thiol-rich domain of KEAP1 [80,81] in a covalent way, either by oxidation or alkylation. These electrophile adducts can inhibit KEAP1 in two different ways, namely: (1) by promoting a conformational change in KEAP1 that will inhibit his binding with NRF2, or (2) by blocking the interaction between KEAP1 and Cul3/Rbx1, inhibiting NRF2 ubiquitination and promoting its sequestration and the subsequent stabilization of the newly synthetized NRF2 [82,83].

In the past years, many natural and synthetic compounds have been described to potentially activate the NRF2 pathway through this mechanism, and these are summarized below.

##### Natural and Semisynthetic NRF2 Activators and Their Analogues

**Figure 7:**
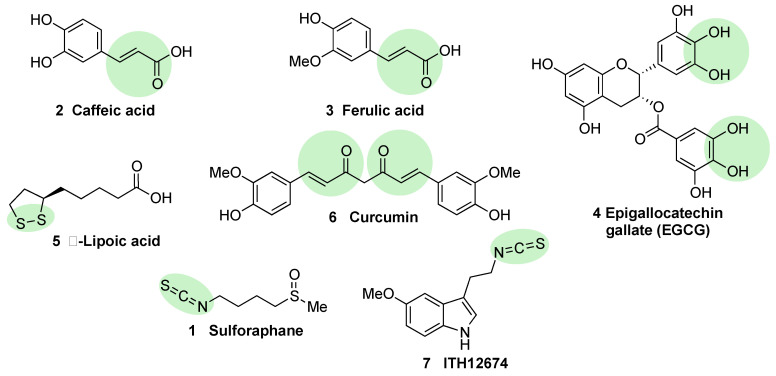
Chemical structures of natural NRF2 activators and their analogues. Caffeic acid (**2**), ferulic acid (**3**), and curcumin (**6**) are considered classical Michael acceptors where a double bound is conjugated with a carbonyl group, creating a potent electrophile. Epigallocatechin gallate (**4**) is a polyphenolic compound that is able to generate quinone derivatives after oxidation, and the corresponding quinone exerts a potent electrophilic character. Lipoic acid (**5**) is considered an electrophile that is able to react with KEAP1 Cys residues to form lipoylcysteinyl disulfides, thus modifying their structure. Finally, isothiothianate moieties at sulforaphane (**1**) and its hybrid melatonin derivative, ITH12674 (**7**), are responsible for the potent electrophilic character of these compounds. The electrophilic moieties or promoieties of each compound are highlighted in green.

Caffeic acid (**2**, Figure 7) is a polyphenol present in coffee bearing a Michael acceptor in its structure. Some studies reveal that this structure is responsible and essential for its NRF2 induction properties while its nucleophilic moiety (catechol moiety) provides clearance but it is not directly involved in NRF2 induction [84]. Further studies have shown that caffeic acid is also able to decrease KEAP1 expression to activate NRF2, leading to an increase in the expression of HO-1 (heme oxygenase 1) and NQO1 (NAD(P)H:quinone oxidoreductase 1) [84].

Ferulic acid (**3**, Figure 7) is highly present in vegetables, and its main structural difference with caffeic acid is one methoxy group on its benzene ring. It has been proved that ferulic acid is able to regulate the NRF2 pathway and counteract trimethyltin (TMT)-induced neuronal damage in the human neuroblastoma cell line SH-SY5Y [84]. Moreover, it is able to antagonize oxidative stress by ROS scavenging and activating the non-homologous end-joining DNA repair process [84].

Epigallocatechin gallate (EGCG) (**4**, Figure 7), a catechin found in green tea [85], upregulates the NRF2 pathway through electrophilic disruption via its prior auto-oxidation of its catechol moieties to ortho-quinones [86], as well as via activation of the p38-MAPK and extracellular signal-regulated kinases (ERK)1/2 signaling pathways [87]. EGCG has shown multiple in vivo and in vitro neuroprotective effects in different models of MS, PD, and traumatic brain injury, all of them associated with an increase in NRF2 induction, antioxidant activity, and a decrease of inflammatory responses [88,89,90].

Alpha lipoic acid (**5**, Figure 7) can be found in a wide number of plants including carrots, beets, broccoli, and spinach. The exact mechanism through which it induces NRF2 is not currently known, although in view of its structure, it can form lipoylcysteinyl disulfides with KEAP1, preventing NRF2 degradation [91,92]. Besides this potential mechanism, it has also been shown that alpha lipoic acid can activate protein kinase C (PKC), which is one of the kinases that is able to activate NRF2 through an alternative pathway [93]. Several studies on alpha lipoic acid neuroprotective effects have been carried out, although in many of them, it was not investigated whether NRF2 activation took part in these effects. In mice MS models, alpha lipoic acid reduced inflammation [94]. In addition, in PD models, it decreased ROS, restored ATP levels, preserved dopaminergic neurons, and upregulated mitochondrial formation [95].

Curcumin (**6**, Figure 7) is the main curcuminoid present in turmeric acid; it has potent antioxidant and anti-inflammatory properties [96,97]. It activates NRF2 by electrophilic modification of Cys-151 in KEAP1, modifying also its ROS-scavenging activity [98]. On the other hand, curcumin is also able to activate NRF2 by repressing KEAP1 expression [99]. Curcumin showed beneficial neuroprotective activities in intracerebral hemorrhage as well as in traumatic brain injury models [25,100]. A decreased in proinflammatory gene expression through the prevention of NF-kB activation in microglial cells was also observed upon treatment with curcumin [101].

Sulforaphane (**1**, Figure 7) is an isothiocyanate produced from the enzymatic cleavage of the organosulfur compound glucoraphanin found in cruciferous plants such as broccoli, brussels sprouts, cauliflower, and cabbage [1]. The catalytic reaction needed for the release of sulforaphane is driven by a β-thioglucosidase present in the gut microbiota [102]; thus, the levels of released sulforaphane are highly dependent on dietary habits and microbiome composition [102]. Sulforaphane directly interacts with the Cys151 residue in KEAP1, thereby promoting NRF2 activation [103,104]. Either in pure form or as sprout extract, sulforaphane has shown a very good safety profile, and more than 30 clinical studies against chronic diseases have been carried out [105,106]. It has the ability to cross the blood–brain barrier (BBB), and it has shown neuroprotective capacity against Aβ_1-42_ peptide in neuronal cells [107]. In vivo, sulforaphane alleviates cognitive impairment in mouse models of AD [108]. In PD models, it protected dopaminergic neurons against the parkinsonian toxin 6-hydroxydopamine [109]. Furthermore, sulforaphane is able to reduce the levels of phosphorylated tau and increase Beclin1 and LC3-II, leading to the conclusion that NRF2 activation may facilitate tau degradation through autophagy [56]. Nevertheless, sulforaphane is an oily substance with low stability in hydrophilic media, and its pharmacokinetic properties need to be improved [1]. In this regard, sulforadex (SFX-01), a sulforaphane-β–cyclodextrin complex, has shown an excellent bioavailability and is under clinical trials for the treatment of subarachnoid hemorrhage and metastatic breast cancer [1]. We will finally mention that sulforaphane has been hybridized with the natural antioxidant melatonin generating ITH12674 (**7**, Figure 6), which is a compound designed to have a dual drug–prodrug mechanism of action for the treatment of brain ischemia [110].

**Figure 8:**
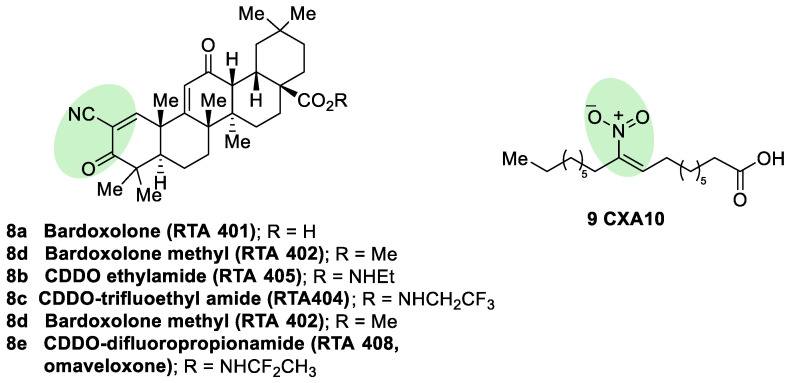
Chemical structures of some semisynthetic cyanoenone triterpenoids (**8**) and a nitro fatty acid (**9**). These compounds are considered electrophilic NRF2 inducers due to the presence of α,β-unsaturations conjugated with carbonyl, cyano, or nitro groups. These electrophilic moieties are highlighted in green.

Semisynthetic cyanoenone triterpenoids derived from bardoxolone (RTA401, CDDO, **8a**, Figure 8), described as antioxidant inflammation modulators (AIMs), exhibit a Michael acceptor moiety and are among the most potent known electrophilic NRF2 activators [111], which interact with Cys151 in KEAP1 and are under clinical development by Reata Pharmaceuticals and Kyowa Hakko Kirin. Proof-of-concept studies strongly support the use of these triterpenoids for degenerative diseases [111]. For instance, CDDO-ethyl amide (RTA405, **8b**, Figure 8) and CDDO-trifluoethyl amide (RTA404, **8c**, Figure 8) induced a significant reduction of toxicity levels in PD models [65]. CDDO-methyl ester (CDDO-Me, RTA402, **8d**, Figure 8) was the first CDDO to reach clinical trials for the treatment of diabetic nephropathy, although it was later withdrawn at phase III (BEACON trial) due to cardiovascular safety issues that are not related with NRF2 induction [112]. In an effort to improve the safety profile, CDDO-difluoropropionamide (RTA408, omaveloxone, **8e**, Figure 8) was synthetized and is currently in phase II trial for the treatment of FRDA, ocular inflammation, and pain after ocular surgery [113].

Nitro fatty acids (NO_2_-FAs) are endogenous signaling mediators with anti-inflammatory and antifibrotic activities in preclinical animal models of metabolic and inflammatory disease [114]. The nitro alkene structure confers electrophilicity to their β-carbon, enhancing the formation of Michael adducts with nucleophiles such as cysteines. It has been proven that NO_2_-FAs react with Cys-273 and Cys-288 of KEAP1, activating NRF2 [115]. The reversibility of this reaction [116] prevents the formation of stable adducts, which could lead to toxicity. Furthermore, compound **9** (10-NO_2_-OA, CXA10, Figure 8) proved to be safe in humans at an active dose in a phase I safety assay [117], and it is currently being tested for the treatment of focal segmental glomerulosclerosis.

##### Synthetic NRF2 Activators

**Figure 9:**
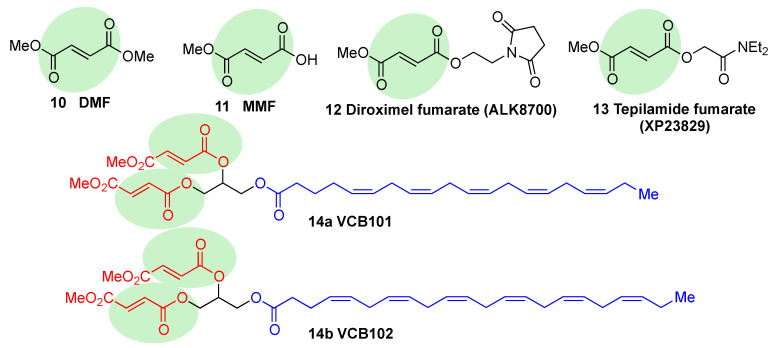
Chemical structures of fumaric acid ester derivatives. Their electrophilic moieties are highlighted in green.

Fumaric acid esters are the group of synthetic NRF2 activators that are receiving the highest amount of attention from the pharmaceutical industry [20]. Dimethyl fumarate (DMF) (**10**, Figure 9) is the most clinically successful member of this family. It was firstly approved in 1994 for the treatment of psoriasis [118], but due to its efficacy in multiple sclerosis mouse models, it was repurposed in 2013 by Biogen under the name Tecfidera for the treatment of relapsing-remitting MS [119], and it has become one of the most successful new medicines in the past few years [1]. Due to its thiol-reactive Michael acceptor structure, DMF primarily activates NRF2 via cysteine modification on KEAP1 [120], and it has also been proven to affect NRF2 phosphorylation via the phosphatidylinositol-3-kinase (PI3K) and ERK1/2 pathways [121].

DMF has shown to be protective in a wide range of neurodegenerative conditions in an AD model [122] and to prevent hippocampal injury after ischemia and protect BBB integrity in mouse models of stroke [123]. Furthermore, protection against α−Syn and Aβ toxicity and a reduction in tau hyperphosphorylation [58,124,125] was also observed.

DMF is mostly converted into monomethyl fumarate (MMF, **11**, Figure 9) by intestinal esterases. Moreover, it has been shown that MMF reacts with Cys151 in KEAP1, thereby activating NRF2 [126]. Several biopharmaceutical companies are developing slow-release forms of MMF in order to improve its bioavailability and reduce the side effects associated to DMF [111]. Thus, Alkernes has developed ALK8700 (diroximel fumarate, **12**, Figure 9), an MMF prodrug with reduced side effects that is now in phase III trials for MS [127]. Tepilamide fumarate (XP23829, **13**, Figure 9) is another MMF prodrug, which has been developed by XenoPort [20]. This compound has shown better solubility and permeability compared to DMF, and also an improved efficacy and reduced gastrointestinal side effects, and it is now in phase II clinical trials for the treatment of plaque psoriasis [20]. A conjugate of MMF and docosahexaenoic acid (CAT4001) is being developed by Catabasis Pharmaceuticals. In animal models, it has shown promising activities for the treatment of NDDs, such as FRDA and ALS [20]. With a similar design, V ClinBio developed conjugates built around central glycerol unit and containing two molecules of MMF linked to one of eicosapentenoic acid (VCB101, **14a**, Figure 9) or docosahexenoic acid (VCB102, **14b**, Figure 9) for the treatment of MS and psoriasis [20].

**Figure 10:**
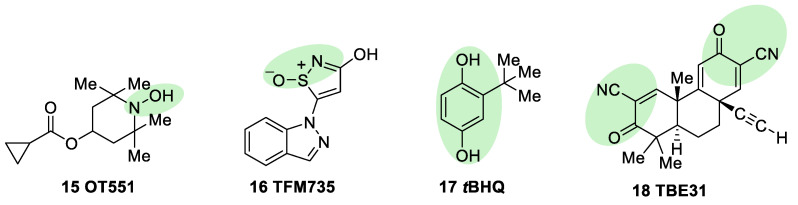
Chemical structures of OT551, TFM735, *t*BHQ, and TBE31. Their electrophilic moieties or promoieties are highlighted in green.

OT551 (**15**, Figure 10) is an *N,N*-disubstituted hydroxylamine developed by Othera Pharmaceuticals [1,128]. It is a prodrug whose active metabolite TEMPOL (4-hydroxy TEMPO, 4-hydroxy-2,2,6,6-tetramethylpiperidine 1-oxyl) inhibits oxidative stress and disease-associated inflammation by a complex mechanism that, besides its radical scavenging activity, involves a reduced activation of NF-κB [129] in acute inflammation and activation of the KEAP1–NRF2 pathway, both indirectly and by targeting KEAP1 [130]. It was designed for topical use and protected the retinal pigment epithelium and photoreceptors from oxidative damage and inflammation in preclinical studies. It has also shown efficacy in a phase II clinical trial on age-related macular degeneration [20].

During a high-throughput screening study, TFM735 (**16**, Figure 10) was identified as an activator of NRF2 through a Cys151-dependent mechanism [131]. This compound is currently in preclinical development by Mochida Pharmaceuticals for the treatment of MS [20].

*t*BHQ, *tert*-butylhydroquinone (**17**, Figure 10) is a metabolic precursor of an electrophilic quinone that is able to disrupt the KEAP1/NRF2 complex. It is widely used as a food preservative and due to its antioxidant properties, it has been also employed with neuroprotective purposes. Treatment with *t*BHQ reduced oxidative stress, and it also prevented neuronal toxicity and Aβ formation in NT2N cell lines [132]. Furthermore, it also led to a decrease in secondary injury and an improvement in function recovery after traumatic brain injury [133].

TBE-31 (**18**, Figure 10), a fully synthetic acetylenic tricyclic bis(cyanoenone), is one of the most potent known NRF2 inducers [91]. It has been mainly studied in cancer models, presenting strong antioxidant and anti-inflammatory activities. Furthermore, treatment with TBE-31 improved mitochondrial function and protection against oxidative stress in fibroblasts and cerebellar granule neurons in FRDA models [134].

#### 5.1.3. KEAP1 Protein–Protein Interaction Inhibitors

KEAP1 is the main regulator of NRF2, by promoting its degradation [8]. The NRF2 recognition by the Kelch domain included in KEAP1 is a classic example of a protein–protein interaction as a drug target, which, generally speaking, remains a challenge for drug discovery due to the large surface of the targeted area and the lack of pockets for the interaction with inhibitors. However, in the case of KEAP1, the target surface is about 300–1000 Å^2^ in size and has well-defined pockets with residues capable of stably recognizing small molecules [135]. These topological characteristics, together with the availability of KEAP1 3D structures, allow the development of small molecules that are able to recognize KEAP1 and disrupt its binding with other proteins [136].

**Figure 11:**
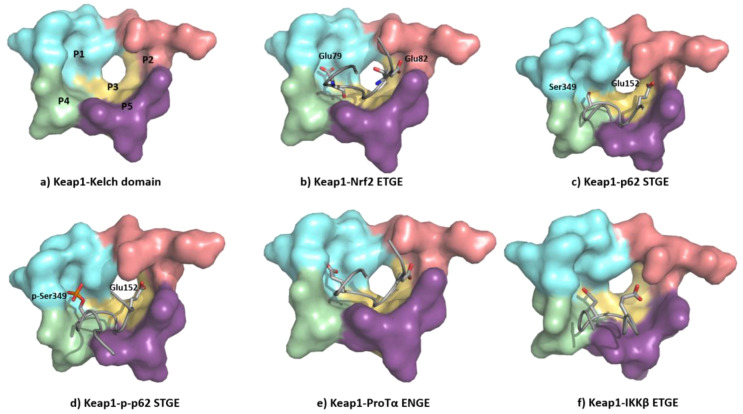
(**a**) Structure of the Kelch domain of KEAP1 (PDB: 5WTV), highlighting binding pockets P1: blue (residues 415, 461, 462, 478, 483 and 508); P2: red (363, 380, 381 and 414); P3: yellow (364, 509, 556, 571, 602 and 603); P4: green (334, 572 and 577); P5: purple (525, 530 and 555); (**b**) structural detail of the NRF2 ETGE motif bound to KEAP1 (PDB: 5WTV); (**c**) structural detail of the p62 STGE motif (PDB: 3ADE). The STGE motif in the KEAP1-interacting region (KIR) is similar to the ETGE-like motif of NRF2 and they both adopt similar β-turn conformations. However, while Glu79 in ETGE interacts with the Arg415 contained in the KEAP1 P1 subpocket, the corresponding Ser349 in STGE is too short, leading to a poor binding affinity; (**d**) structural detail of the Ser349-phosphorylated p62 STGE motif (PDB: 3WDZ). Phosphorylated Ser439 is deeply inserted into the P1 subpocket, and the negative charges increase the affinity through electrostatic interactions with Arg415 and Arg483; (**e**) structural detail of Protα ENGE motif (PDB 2Z32); (**f**) structural detail of the IKKβ ETGE motif obtained by molecular dynamics [149]. The substitution of the Thr residue (DEETGE, NRF2) by an Asn residue (NEENGE, ProTα) renders the β-turn conformation unstable, leading to a less potent binding between the ENGE motif and KEAP1 compared with that of the ETGE motif in NRF2.

The ETGE motif contained in the Neh2 domain of NRF2 adopts a β-hairpin conformation to interact with the KEAP1 Kelch domain-binding pocket, which can be artificially subdivided into five subpockets (Figure 11a) [136]. The P1 and P2 subpockets are characterized by the presence of multiple positively charged arginine residues (Arg415, Arg483, and Arg485), enabling these sites to recognize negatively charged amino acid residues of substrates [137]. In particular, the carboxyl side chains of Glu79 and Glu82 at the ETGE motif of NRF2 (Figure 11b) promote binding by their interaction with the P1 and P2 subpockets. The central P3 subpocket is formed by small polar and nonpolar residues that stabilize the ETGE motif. The external P4 and P5 subpockets are formed by aliphatic chain and tyrosine-rich residues and improve the substrate recognition through hydrophobic interactions and hydrogen bonds [136,137]. 

The Kelch domain in KEAP1 is able to interact with several additional proteins containing ETGE-like sequences in a similar manner to NRF2 [138]. Some of them, such as SQSTM1/p62 and prothymosin α (ProTα), are also related to the regulation or the NRF2–ARE pathway, or they are implicated in the crosstalk with other hallmarks of neurodegeneration, such as IKK-β (inhibitor of the nuclear factor kappa-B kinase subunit beta) in neuroinflammation [138].

The KEAP1 Kelch domain recognizes p62 through a KEAP1-interacting region (KIR) and this disrupts the KEAP1–NRF2 system, leading to a crosstalk between the NRF2–ARE pathway and p62 [139] (Figure 11c). The mTORC1-dependent phosphorylation of this Ser349 elongates the side chain and adds negative charges to it, improving its recognition by KEAP1 (Figure 11d) [140]. The p62 protein is a cargo receptor for selective autophagy, and its strong interaction with KEAP1 allows it to sequester the latter into early autophagosomes, promoting its degradation through the autophagy–lysosome pathway [141]. Both the p62-mediated disruption of the KEAP1–NRF2 interaction and the p62-dependent degradation of KEAP1 increase the stability of NRF2, leading to neuroprotection [142]. Another ETGE-like substrate that can bind KEAP1 is ProTα, whose ENGE motif mediates its interaction with the KEAP1 Kelch domain (Figure 11e). The crystal structure of the KEAP1–ProTα complex shows a similar network of interactions to the NRF2 ETGE motif [143]. ProTα exhibits a neuroprotective profile against ischemia–reperfusion injury, which can be explained by its capacity to disrupt the NRF2–KEAP1 complex when present in a large excess [144,145].

KEAP1 also interacts with IKKβ, which plays a central role in the control of the pro-inflammatory NF-κB pathway after phosphorylation and several downstream steps [146]. The recognition between KEAP1 and IKKβ promotes the degradation of the latter and prevents the phosphorylation of IKKβ, reducing neuroinflammation [147]. The interplay between the NRF2–ARE and NF-ĸB pathways seems especially interesting in neurodegenerative and cerebrovascular diseases and can be explained in part by the interaction of KEAP1 with NRF2 and IKKβ [147]. Although a crystal structure of the KEAP1–IKKβ complex is unavailable [148], it has been studied through protein–protein docking and molecular dynamic analysis, which show that the ETGE motif of IKKβ adopts a β-turn conformation [148], occupying the substrate-binding pocket in a similar fashion to that of the NRF2 ETGE motif (Figure 11f) [149].

**Figure 12:**
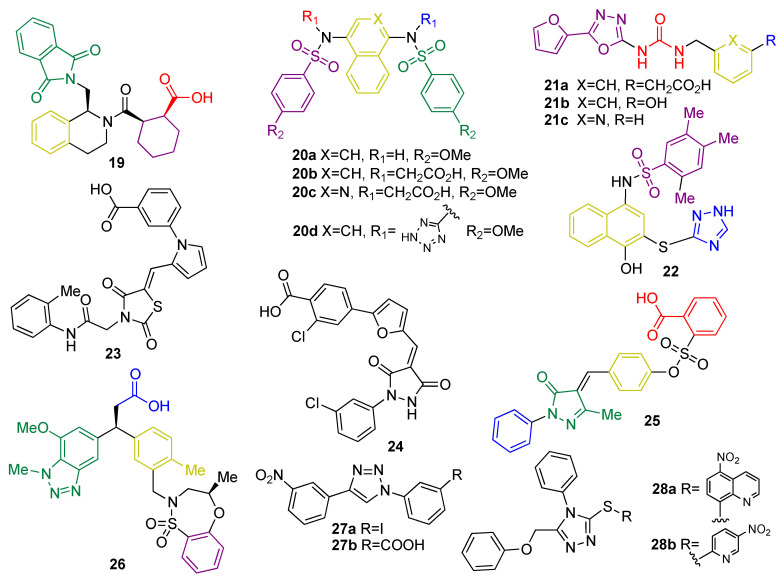
Chemical structures of KEAP1 protein–protein interaction inhibitors **19**–**28**. Compounds **19**, **20**, **21**, **22**, **25** and **28** have been crystalized with KEAP1 and/or their interaction pattern has been proposed by using molecular modeling and molecular dynamics. Their interaction pattern with KEAP1 has been coded by giving different colors to the motif of each molecule that interacts with each pocket as follows: interaction at P1: blue; interaction at P2: red; interaction at P3: yellow; interaction at P4: green; and interaction at P5: purple.

Small peptides based on structural similarity to KEAP1 substrates were the earliest examples of molecules designed to act as KEAP1 inhibitors [150]. However, they have several limitations, including their inability to cross the BBB and their poor stability. Thus, the development of small molecules as KEAP1 binding disruptors remains the main approach, overcoming the bioavailability and stability issues associated to peptides [151]. HTS of a library of more than 300,000 compounds led to tetrahydroisoquinoline **19** (Figure 12) as the first-in-class small-molecule KEAP1–NRF2 PPI inhibitor [151]. Further research efforts on this scaffold led to new structure–activity relationships [152]. The naphthalene sulfonamide derivative **20a** (Figure 12) was also identified as a KEAP1–NRF2 inhibitor in a fluorescence anisotropy assay [153]. Structural determination showed an interaction between **20a** and the P3–P5 subpockets in the Kelch domain of KEAP1. Based on this finding, compound **20b** (Figure 12) was designed [153] with its two carboxyl groups forming multiple hydrogen bonds and electrostatic interactions with the basic Arg residues present in P1 and P2, thus improving the affinity with the KEAP1–NRF2 interface [153]. In the development process, the naphthalene core was replaced by an isoquinoline core to give **20c** (Figure 12), improving the solubility, metabolic stability, and reducing the intrinsic toxicity of naphthalene scaffold [154]. Recently, the exploration of bioisosteric replacements of both carboxylic acids afforded the ditetrazole analogue **20d** (Figure 12), which maintains the potent PPI inhibition activity and improves the cellular potency thanks to a better pharmacodynamic profile [155]. Another study demonstrated that oxadiazole-urea based compounds **21a-c** (NK-252, Figure 12) interact with the KEAP1–Kelch domain [156], with the carboxylic group and the urea–oxadiazole structure interacting via hydrogen bonds and the phenyl moiety by π-stacking with the P1, P2 and P3 subpockets, respectively.

Virtual screening of a 300,000-compound library afforded several hit structures that are able to interact with the KEAP1–NRF2 interface, including compounds **22**, **23**, and **24** [157]. Compound **25**, structurally related to **20**, exhibits a very interesting profile as a neuroprotector in a PD animal model by inhibiting the KEAP1–NRF2 interaction [158]. Fragment-based drug discovery was successfully employed in the development of **26**, which is a novel phenylpropanoic acid-based KEAP1–NRF2 PPI inhibitor [159]. Crystallographic studies revealed the overlapping between the original fragments and **26** in the binding pocket [159]. This compound showed potency in cell-based assays in the nM range and was shown to activate the NRF2 pathway in vivo [99]. Similarly, simpler five-membered heterocycles such as 1,4-diphenyl-1,2,3-triazoles **27a-b** were designed *in silico* to mimic the Glu residues in NRF2 and were shown to interfere in the KEAP1–NRF2 recognition process and induce the expression of NRF2 target genes in live cells [160]. Isomeric compounds containing the 1-phenyl-1,3,4-triazole scaffold were also described in the patent literature [161]. Compounds **28a** and **28b** (Figure 12) inhibit the KEAP1–NRF2 interaction with moderate inhibitory potency and induce the expression of NRF2 downstream target genes, leading to neuroprotection in an in vitro model of PD [161]. In particular, compounds **28** are particularly promising as anti-neurodegenerative agents, since in vivo studies using a HD model revealed that they inhibit the neurodegeneration of medium spiny neurons [162].

Research into KEAP1–NRF2 PPI inhibition is rapidly becoming a hot topic in medicinal chemistry, and it is evolving rapidly. In this context, the availability of crystal structures and a broad range of structural analyses related to recognition processes at the KEAP1–NRF2 complex have contributed to the development of several lead compounds by modern drug discovery strategies and the exploration of new chemical sources [152,157,161]. However, these molecules are often polar and possess a relatively large molecular weight, with a limited BBB penetration, leading to poor pharmacodynamic and pharmacokinetic profiles in NDDs [136]. Therefore, PPI inhibitor design with high potential activity and improved physicochemical properties is crucial to develop a new generation of drugs with a better safety profile, compared to KEAP1 covalent inhibitors.

#### 5.1.4. Epigenetic Control of KEAP 1 Expression

**Figure 13:**
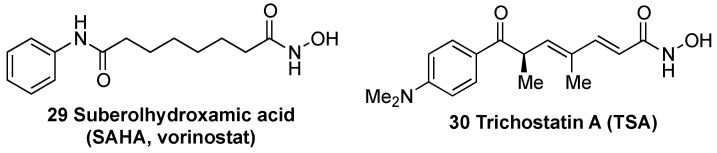
Chemical structures of histone deacetylases (HDAC) inhibitors suberolhydroxamic acid (SAHA) (**29**) and trichostatin A (TSA) (**30**).

Histone deacetylases (HDACs) remove acetyl groups in histones associated to the KEAP1 promoter region, inducing an increase in KEAP1 transcription [163]. Consequently, HDAC inhibitors, which counteract this effect, improve the redox balance and attenuate neuronal degeneration in some NDDs such as HD [163], ALS [164], and in animal models of stroke [165]. In a model of transient cerebral ischemia, treatment with suberolhydroxamic acid (SAHA) (**29**, Figure 13), also known as vorinostat, reduced infarct volume by 30–40% [166]. Trichostatin A (TSA) (**30**, Figure 12) was identified as an inhibitor of KEAP1 expression and leads to an improvement of NRF2 activity and protection against cerebral ischemia [167].

MicroRNAs (miRNAs or miRs) are small noncoding RNAs with 18–25 nucleotides in length that are able to bind to the 3′-untranslated region (UTR) of the target mRNAs [168]. After miRs and target mRNA binding, and depending on the complementarity of both sequences, the target mRNA is degraded or its transcription is suppressed [168]. The miR-7 reduce the expression of KEAP1 after the interaction with its 3′-UTR in the SH-SY5Y cell line [169]. This downregulation of KEAP1 expression leads to NRF2 stabilization and increases the expression of several cytoprotective proteins. The effect of miR-7 appears to be especially interesting in PD models, where the overexpression of miR-7 protects neuronal cells against oxidative stress [169]. It is also relevant that the brain regions more affected in PD, such as *substantia nigra* and *striatum*, show an increased level of miR-7 compared with non-affected regions such as the cortex or cerebellum [170]. Furthermore, miR-7 expression downregulation observed in a PD mice model [170] suggests its implication in PD development.

Based on these precedents, KEAP1 expression regulation by epigenetics or miRs can provide an effective strategy to improve neuroprotection through the NRF2–ARE pathway.

### 5.2. KEAP1-Independent Regulation

As discussed above, NRF2 activation is mainly regulated by KEAP1, which is a mechanism known as the canonical pathway. However, many non-KEAP1-related processes contribute to NRF2 regulation and are summarized in this section.

#### 5.2.1. NRF2 Regulation by GSK-3β

GSK-3β is a Ser/Thr kinase involved in glycogen metabolism, cell proliferation, apoptosis, and other functions within the cell [171]. Regarding NDDs, GSK-3β activity has been reported to be upregulated in AD and has been considered to play a key role on tau hyperphosphorylation [172]. Therefore, GSK-3β has been one of the most important targets in drug development toward AD. In 2006, Rojo et al. described the ability of this kinase to modulate the NRF2–ARE response [173], promoting NRF2 phosphorylation and nuclear exclusion mediated by Fyn [174]. GSK-3β phosphorylates Fyn, to increase its activity; in turn, Fyn phosphorylates NRF2 at Tyr568, leading to its extranucleation [175] and degradation.

Additionally, another line of research has demonstrated that GSK-3β phosphorylates the DSGIS [17] degron motif at Neh6 of NRF2 [176], as described above. This post-translational modification increases the affinity of β-TrCP for NRF2 to induce its proteasomal degradation [17], and it is further discussed in next paragraph. Moreover, GSK-3β its activity is regulated by several kinases: the PI3K/Akt pathway downregulates GSK-3β by the phosphorylation of Ser9 [177] to induce its inactivation [178], and p38-MAPK also regulates its activity by phosphorylation at Thr390 [179].

As summarized in Table 1, the GSK-3β regulatory mechanism of NRF2 has renewed the interest of this important kinase as a potential target for NDDs, with special emphasis in AD.

Several GSK-3β inhibitors have reached clinical trials toward different diseases including AD, mild cognitive impairment, autism spectrum disorders, and cancer. Initially, NRF2 activation via GSK-3β inhibition was demonstrated for LiCl, a GSK-3β inhibitor used for the treatment of bipolar disorder and compound TDZD-8 [180], which is able to increased HO-1 expression. Recently, LiCl-induced NRF2 activation has been reported in different in vitro and in vivo models, confirming the regulation of the latter by GSK-3β [181]. TDZD-8-induced NRF2 activation has been further demonstrated in an in vivo model of renal ischemia/reperfusion injury. It reduced oxidative stress and cellular apoptosis via NRF2 activation [182].

A clinically advanced example is tideglusib, a thiadiazolidinone derivative, which was developed as a non-ATP competitive GSK-3β inhibitor for AD treatment, which reached phase II clinical trial (ARGO study) [183], but it failed to produce clinical benefit. Researchers suggested a potential negative effect of the non-linear dose response and propose further dose-finding studies in early AD cases. Tideglusib and pioglitazone, also a thiadiazolidinone derivative, showed neuroprotection against MPPT toxicity mediated by NRF2 induction and phase II response activation. In this model, authors demonstrated that the activation of the antioxidant response was mediated by GSK-3β inhibition [184], although the neuroprotective effect was observed at a higher concentration (5 μM) compared to its GSK-3β IC_50_ (5 nM).

Another example of the NRF2 induction capacity of GSK-3β inhibitors is compound SB216763, which is a highly potent and selective GSK-3β inhibitor. SB216763 showed an interesting cytoprotective effect against the toxicity induced by doxorubicin in primary podocytes via the pathological activation of GSK-3β and oxidative stress [185]. SB216763 protected podocytes through NRF2 induction and phase II antioxidant response activation, and this response was directly associated to its GSK-3β inhibition capacity [185]. Finally, NRF2 induction via GSK-3β inhibition has been demonstrated for many other GSK-3β inhibitors such us YQ138 [186], obacunone [187], or corilagin [188]; however, in the latter cases, the NRF2 mechanism of action can also be related to a direct interaction with KEAP1 or other activation pathways due to the structural features and multi-target activity of the compounds.

#### 5.2.2. KEAP1-Independent Regulation of NRF2 Stability

**Figure 14:**
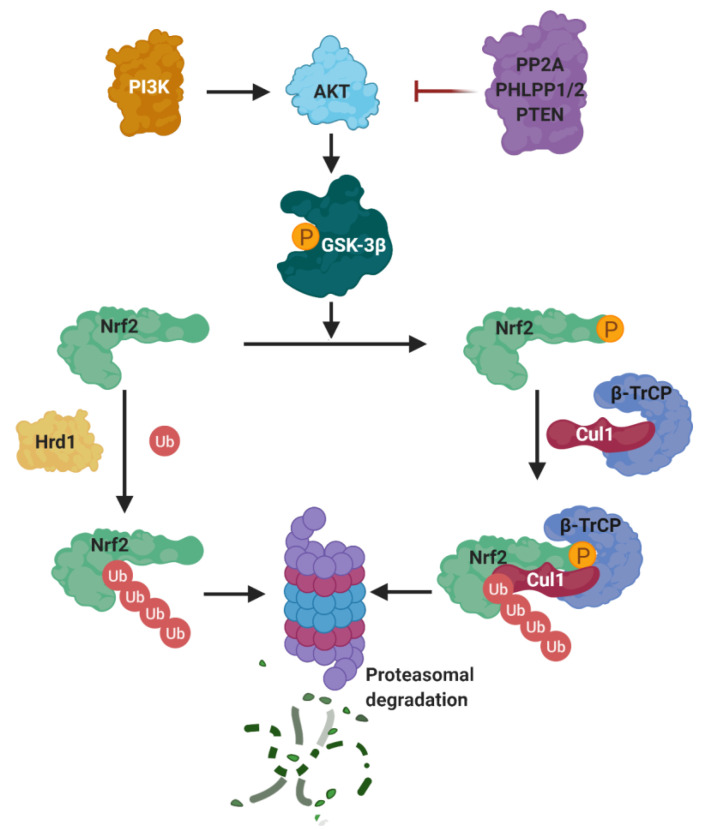
KEAP1-independent negative regulation of NRF2. The latter protein is tightly regulated by different systems, independently of its principal repressor KEAP1, to ensure the activation of the phase II response only under stress conditions. From left, the activation of different pathways might lead to PI3K activation that activates AKT by phosphorylation that in turn, from right, was inhibited by protein phosphatase 2A (PP2A), PHLPP1/2 (PH domain and leucine rich repeat protein phosphatases 1 and 2), or tumor suppressor phosphatase and tensin homolog (PTEN). After its activation, AKT is able to phosphorylate GSK-3β at Ser^9^ inhibiting its activity reducing the negative regulation of NRF2 by this kinase. GSK-3β is known to act as a negative NRF2 regulator by several mechanisms. NRF2 can be phosphorylated by this kinase at Ser335 and S338 to create a phosphodegron domain DSGIS recognized by β-TrCP that leads to Cul1/Rbx1 NRF2 ubiquination and proteasomal degradation. NRF2 is also regulated by a third ubiquitination-degradation system by interaction with Hrd1, at the Neh4 transactivation domain, which is a ubiquitin ligase that targets NRF2 for proteasomal degradation.

Besides KEAP1, there are other mechanisms that control the stability of NRF2. The most important ones are the β-transducin repeat containing protein (β-TrCP), connected to the PI3K/AKT pathway, and Hrd1 (Figure 14).

*(a) PI3K/Akt*. The PI3K/Akt pathway regulates metabolism, growth, proliferation, cell survival, and gene transcription. Akt is a Ser/Thr kinase activated by several signals that produce phosphatidylinositol-(3,4,5)-trisphosphate (PIP3) catalyzed by phosphatidylinositol-3-kinase (PI3K) [189]. PIP3 serves as anchor for Akt and its activator kinase, PDK1, which activates Akt by phosphorylation at Thr308. Phosphorylated Akt is further activated by mTORC2 by phosphorylation at Ser473 [189]. A negative regulation of Akt is exerted by protein phosphatase 2A (PP2A), PHLPP1/2, and the tumor suppressor phosphatase and tensin homolog (PTEN) [189].

The PI3K/Akt pathway contributes to Nrf2 induction in a KEAP1-independent mode, as described by Cuadrado et al. [190]. By using the PI3K inhibitor LY294002, they observed a substantial inhibition of carnosol-mediated Nrf2 induction. Further evidence to support the regulation of Nrf2 induction by PI3K activation was obtained by using a biotinylated analogue of CDDO-Im, which is a potent Nrf2 inducer belonging to the cyanoenone triterpenoid family that activates the PI3K/Akt pathway because it reacts covalently with Cys124 inside the active site of PTEN [191], inhibiting its activity. Similarly, 4-hidroxynonenal (an Nrf2 inducer) modifies PTEN Cys71 [192]. The Cys71 and Cys124 residues of PTEN act as redox sensors and, when oxidized, they form a disulfide bridge inactivating the PIP3 3-phosphatase activity of PTEN and increasing the activity of Akt [193]. Therefore, PTEN is considered a negative repressor of Nrf2, acting as a redox sensor inside the cell; once it is inactivated, Nrf2 is activated via the PI3K/Akt pathway activation.

It was demonstrated that PI3K activation leads to Nrf2 induction [173,194] by increasing Akt activity that, in turn, inhibits the activity of GSK-3β by phosphorylation at Ser9 [195] and Ser21 [196]. Furthermore, GSK-3β can also be inhibited by PKC [197], P70S6K, and P90RSK [198], all of which are regulated by PI3K through PDK1 [199]. 

*(b) β-TrCP*. The PI3K/AKT pathway activates NRF2 via the inhibition of GSK-3β, which phosphorylates NRF2 [190], allowing its recognition by β-TrCP, which in turn marks NRF2 for ubiquitinylation and subsequent proteasomal degradation [200]. β-TrCP is an F-Box-containing protein of the WD40 subfamily that acts as substrate adaptor/receptor within the Skp1-Cul1-F-box (SCF) ubiquitin ligase SCF^β-TrCP^ [201] and participates in the ubiquination of IκB, β-catenin, CDC25, and APC [70], among others, besides NRF2. β-TrCP recognizes its substrates by direct binding to phosphorylated destruction motifs; for instance, it binds to phosphorylated NRF2 at its ^343^DSGIS^347^ and ^382^DSAPGS^387^ sequences [202]. Interestingly, this sequence mimics the GSK-3β target motifs, and thus many of the GSK-3β target proteins are known to be processed by β-TrCP for degradation [203].

By using selective GSK-3β inhibitors or siRNAs targeting GSK-3β, a stabilization and nuclear localization of NRF2 was initially observed. Similar results were obtained by using siRNAs toward β-TrCP, demonstrating the regulation of NRF2 by both proteins. The Neh6 domain of NRF2 presents two motifs, DSGIS and DSAPGS, that can be recognized by SCF^β-TrCP^ once they are phosphorylated [204]. Thus, the GSK-3β/β-TrCP system induces NRF2 ubiqination via β-TrCP/Cul1in1/Rbx1 E3 ligase complex after phosphorylation, being a KEAP1-independent mechanism activated by receptor signal transduction [205]. Considering this regulatory mechanism, a hypothesis of “dual modulation” by KEAP1 and β-TrCP was proposed by Cuadrado et al. [77]. Therefore, β-TrCP is a fine tune regulator of NRF2 for cell signaling, considering the high number of proteins regulated by Nr2 with a high impact on metabolic adaptation [78].

*(c) DJ-1*. In another connection to NDDs, a direct link between DJ-1, a protein involved in PD, and Nrf2 has been described. DJ-1 inactivation in PD is related to a higher susceptibility of neurons to oxidative stress [206]. Compelling evidence demonstrates that DJ-1 acts as a negative regulator of PTEN [207]. Therefore, DJ-1 downregulation reduces Nrf2 activity due to the increased activity of PTEN. Furthermore, DJ-1 presents several ROS-sensitive Cys residues that can act as oxidative stress sensors. Finally, it has been proposed that DJ-1 can act as a nitric oxide-transferring protein to PTEN Cys residues to inhibit its activity [208].

#### 5.2.3. Miscellaneous ARE Transcription Regulators Connected to NRF2

*(a) AHR.* The aryl hydrocarbon receptor (AHR) is a ligand-activated transcription factor of the bHLH/PAS family that mediates the effects of many xenobiotics (e.g., polyaromatic hydrocarbons, dioxins) and endogenous compounds [209]. It is widespread in the nervous system and, among others, regulates neuronal functions [209]. Miao et al. showed that the transcription of NRF2 is directly regulated by AHR [210], and in fact, they exhibit both ARE and XRE response elements in their promoter region. Moreover, they share several common antioxidant target genes [211].

*(b) P53.* The P53 protein, known as the guardian of the genome, is a transcription factor that is activated by DNA damage, regulating the pathways for cell-cycle arrest, DNA repair, senescence, and apoptosis. Indeed, it has been proposed that p53 may contribute to neuronal death processes common to all NDDs [212]. There are several connections between the NRF2 and p53 pathways [213]. First, the activation of p53 induces the expression of p21, which competes with KEAP1 for binding to NRF2 and prevents NRF2 degradation, rising its levels [214]. Moreover, NRF2 induces the expression of NQO1, which hinders the degradation of p53 by the 20S proteasome [215].

**Figure 15:**
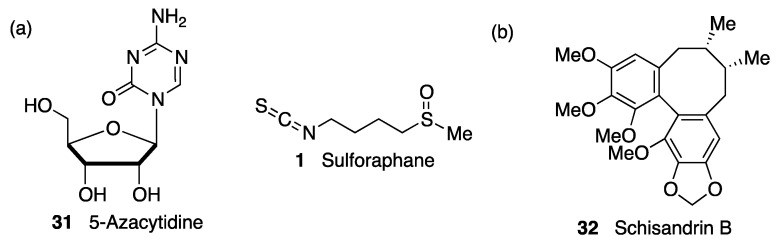
(**a**) Two compounds that exerted neuroprotection by epigenetically-promoted upregulation of NRF2 expression. (**b**) Schisandrin B exerted neuroprotection in a Parkinson’s disease (PD) cell model by blocking the suppression of NRF2 activity by the microRNA miR-34a.

*(c) Epigenetic regulation.* As in the case of KEAP1, the transcription of NRF2 is subject to epigenetic regulation via methylation of the NRF2 promoter in CpG islands, H3 histone methylation, and H4 histone acetylation [216]. There is some evidence showing that DNA-methyltransferase inhibition during brain development increases susceptibility to oxidative DNA damage in the aged brain via the hypomethylation of promoters of AD-associated genes such as the β-amyloid precursor protein [217]. Interestingly, DNA demethylation by treatment with 5-azacytidine (**31**, Figure 15) was shown to upregulate NRF2 expression in an AD cellular model. [218] In the same context, it has been proven that one of the mechanisms of neuroprotection by sulforaphane (**1**, Figure 15) in mouse neuroblastoma cells is an upregulated NRF2 expression and promoted NRF2 nuclear translocation by decreasing the methylation levels of the NRF2 promoter gene [219].

NRF2 activity can also be regulated by microRNAs, which repress protein production by interacting with complementary seed sequences in the UTR of target mRNAs. Several miRs are involved in direct NRF2 regulation and are related with neurological disorders associated with oxidative stress [168]. Thus, miR-27a, miR-142-5p, miR-144, and miR-153 were identified as NRF2 suppressors by *in silico* analysis. Studies in neuronal SH-SY5Y cells showed that the overexpression of these miRs suppresses NRF2 activity by targeting its mRNA, leading to uncontrolled redox homeostasis [220]. Accordingly, schisandrin B (**32**, Figure 15), a dibenzocyclooctadiene lignan found in the traditional Chinese medicinal herb *Schisandra chinensis*, has shown neuroprotective effects in 6-OHDA PD models by acting as an inhibitor of miR-34a action [221]. Many miRs are involved in the pathological events after cerebral stroke, and among them, miR 93 has been found to be a negative regulator of Nfr2 expression that contributes significantly to deregulation of the redox balance in ischemia [222]. On the other hand, miR-424 upregulates NRF2, and its overexpression has an antioxidant effect in focal cerebral ischemia/reperfusion in mice that is suppressed by NRF2 knockdown and treatment with SOD inhibitors [223].

#### 5.2.4. Miscellaneous Pathways Regulating the Post-Translational Phosphorylation of NRF2

Beyond the canonical pathway, the tight control of the phase II antioxidant response inside the cells encompasses a plethora of pathways that regulate NRF2 by different post-translational mechanisms [224,225]. Different positive and negative NRF2 regulation proteins have been described including the previously discussed GSK-3β [173], PI3K/Akt [190], MAPKs [226,227], β-TrCP [190], and PKC [228,229], among others. This fine-tuned control over NRF2 through non-canonical mechanisms opens up the opportunity to design drugs aimed at specific targets.

*(a) MAPKs.* These enzymes are serine/threonine kinases that modulate crucial biological processes including cell survival [226,230], apoptosis [228,231], and gene expression [227,230]. There are three main MAPK pathways composed by the ERKs: the c-jun N-terminal kinases (JNKs) and the p38 kinases directed to phosphorylation of Ser or Thr residues near to Pro residues [227]. NRF2 regulation by different MAPK pathways has been widely described by the use of selective inhibitors and transfection studies, which demonstrated that ERK signaling increases NRF2 activity [232] and P38 reduces NRF2 signaling [233]. Sun et al. demonstrated that a number of phosphorylation sites at NRF2, including Ser215, Ser408, Ser558, Thr559 and S577, are post-transcriptionally modified by the members of this family of kinases [234].

*(b) P38.* The P38 MAPK family comprises several isoforms (P38α, β, γ and δ) with specific distribution and substrates. Once activated by phosphorylation, they in turn phosphorylate different proteins and transcription factors to regulate processes including inflammation, cell division, and differentiation or apoptosis [235]. P38 kinases are mainly activated by oxidative stress [236], which is a pathological hallmark of NDDs. P38 has been related to AD, since it is activated by the Aβ peptide and hyperphosphorylated tau through oxidative stress [237]. In this line, the P38 pathway was described to be overactive in AD brains at initial stages [238]. The general consensus points to a negative regulation of NRF2 by P38 MAPK as shown by Keum et al., who described the active p38 isoforms that are able to phosphorylate the NRF2 protein to reduce its nuclear accumulation [239]. P38 MAPK phosphorylates NRF2 at three Ser residues (Ser215, Ser408, and Ser577), improving its interaction with KEAP1 and thus promoting its degradation [9]. Conversely, p38 activation activated the NRF2–ARE pathway [240,241] after the administration of several xenobiotics, which is an observation that was correlated with the inhibitory activity of P38 on GSK-3β [239]. Most probably, the activity of P38 on NRF2 depends of small modifications of the homeostatic equilibrium, and the final response depends on the stimuli, timing, and cellular type.

*(c) JNK.* The c-jun-NH_2_-terminal kinase (JNK) pathway is activated mainly by stress stimuli and is an important member of the MAPK signaling pathways [242], playing important roles in development, cell growth, inflammation, and apoptosis [243]. JNKs are a family of threonine protein kinases that includes three different isoforms (JNK1, JNK2, and JNK3), the latter of which is specifically expressed in the central nervous system [244] and is considered a key target for NDDs. JNK3 has been found to be activated in AD [245], PD [246], ALS, and stroke [247], among other NDDs. Sustained JNK activation has been widely related to pathological conditions promoting cell damage and death and has also been correlated with decreased NRF2 signaling. Recently, a direct crosstalk between JNK and NRF2 has been revealed in the context of protection against liver injury induced by acetaminophen, which is closely associated to JNK activation [248]. Active JNK (p-JNK) increased NRF2 turnover by direct interaction with the Neh1 domain of NRF2. This interaction allows the phosphorylation of Ser residues at Neh6 degron domain of NRF2. Thus, it was demonstrated that p-JNK phosphorylates Ser335 at DSGIS motif of NRF2, codifying its degradation through a KEAP1-independent mechanism [249].

*(d) ERKs.* The extracellular signal-regulated kinases (ERKs) are a major MAPK subfamily involved in a large number of biological processes. ERK activation has been highly related to cytoprotection, specifically to neuroprotection, linking this kinase pathway to potential targets against NDDs [250]. NRF2 regulation by the ERK pathway has been reported in many different cellular types, and it has been implicated in the mechanism of action of several NRF2 inducers. *t*BHQ, a known NRF2 inducer [132], increases NRF2 stability inducing the activation of the phase II response in a ERK1/2-dependent mechanism, since its NRF2 induction capacity was abolished in the presence of ERK inhibitors [251]. ERK activation was demonstrated to be necessary for NRF2–ARE signaling in response to several other NRF2 inducers such as pyrrolidine dithiocarbamate [252], caffeic acid [253], sulfuretin [254], gallic acid [254], luteolin [255], and butylated hydroxyanisole [256] in HepG2 cells; lico A in RAW 264.7 cells (26576227), or phenylethanoid glycoside in PC12 cells. Some reports suggest a potential direct crosstalk between NRF2 and ERK1/2 by which activated ERK1/2 might be able to directly phosphorylate NRF2 at Ser40, codifying its nuclear stabilization to increase NRF2–ARE pathway activation [257]. This hypothesis is supported by an increase of p-Ser40-NRF2 protein levels measured by Western blot. However, a direct interaction of ERK1/2 and NRF2 has not been fully proven, and other pathways might be contributing such as PKC, JNK, or Akt [257].

*(e) PKC.* This protein is part of a family of serine/threonine kinases related to many cellular signaling responses including apoptosis, survival, and differentiation. Although PKC activation is traditionally considered receptor-dependent, there is also a ROS-dependent activation mechanism [258]. The discovery that phorbol esters (PKC activators) induce the expression of NRF2-related proteins led to hypothesize the regulation of NRF2-phase II response by PKC [228]. It was proved that, together with other kinases [229], PKC phosphorylates Ser40 at the NRF2Neh2 domain, disrupting the KEAP1–NRF2 interaction [259]. Ser40 phosphorylation is necessary for its nuclear translocation, but it does not facilitate its nuclear accumulation [260]. A second NRF2 regulation mechanism by PKC is related to the capacity of several PKC isoforms (α, β_1_, γ > β_2_; not ε) to phosphorylate GSK-3β at inactivation sites [261]. Focusing on NDDs, PKC protein levels and activity was found to be decreased in AD brains [262]. Considering the positive modulation of NRF2 by PKC and other potentially beneficial properties, several PKC activators have been proposed as potential drugs for AD treatment [263].

*(f) CK2.* Casein kinase II (CK2) is a Ser/Thr kinase that acts on more than 300 substrates, participating in the regulation of many different processes including signal transduction, gene transcription, replication processes, and survival pathways, among others. Among the processes modulated by CK2, it regulates several key regulators against cell stress [264]. CK2 is also related to NRF2 modulation, since its sequence presents at least 13 potential phosphorylation positions targeted by CK2 [265]. By using deletional analyses of NRF2, Neh4 and Neh5 domains were identified as CK2 target regions where this kinase is able to phosphorylate several residues, inducing NRF2 nuclear accumulation and pathway activation [265].

*(g) Fyn kinase.* Fyn kinase, a member of the Src family, is closely related to the negative regulation of NRF2. Fyn kinase directly phosphorylates NRF2 at Tyr568 to codify its nuclear export [266]. Tyr568 phosphorylation facilitates NRF2 recognition by exportin1, which is the protein related to NRF2 nuclear elimination [266].

## 6. Conclusions

**Figure 16:**
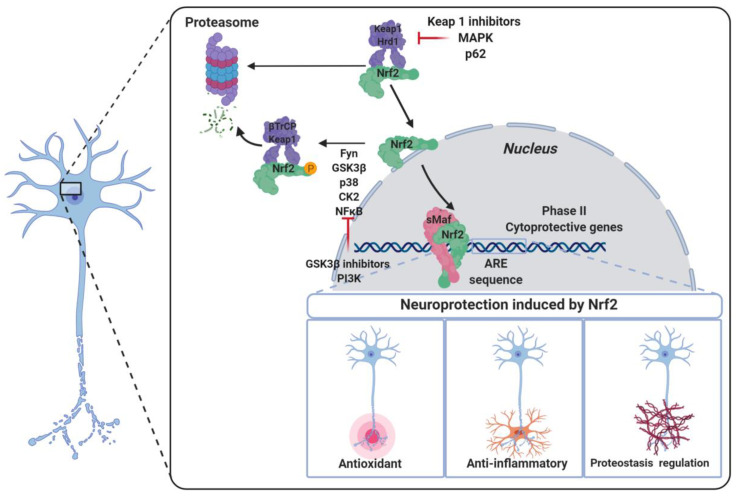
A summary of the main NRF2 regulation mechanisms. KEAP1 represents the main negative regulatory protein of NRF2 coding its proteasomal degradation to maintain its expression at low levels. KEAP1–NRF2 interaction can be blocked by the activity of protein–protein inhibitors, MAPKs and p62. Hrd1 also induces NRF2 proteasomal degradation, helping its regulation. Once activated, NRF2 induces the expression of antioxidant, anti-inflammatory, and proteostasis-related genes promoting neuronal survival. Under pathological conditions, several signals can induce the activation of different kinases and factors that negative regulate NRF2 promoting its degradation. When this intricate regulatory system is dysregulated by a pathological condition, the failure of the NRF2–ARE pathway has been correlated to the advance of the disease.

NRF2 is critical to redox homeostasis and the regulation of neuroinflammation, and an increase in its activity is an interesting approach to the therapy of neurodegenerative diseases. NRF2 regulation takes place at several levels, namely protein stability and transcriptional and post-transcriptional level regulation. Many of the proteins and enzymes involved in these processes provide druggable targets for the treatment of neurodegenerative diseases, as summarized in Figure 16. The modulation of NRF2 regulation is a field that is still in its infancy and is full of opportunities in terms of unexplored potential drug targets, and we hope that this review will stimulate innovative research in this fascinating area.

## Figures and Tables

**Table 1:** GSK-3β inhibitors with NRF2 induction capacity.

Compound	Disease	Clinical Trial	Ref
Pioglitazone 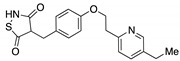	Type II diabetes	Approved	
	Several diseases	300 clinical trials	--
Tideglusib 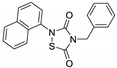	AD	Phase II	NCT01350362
	Congenital myotonic dystrophy	Phase II/III	NCT03692312
	Autism spectrum disorders	Phase II	NCT02586935
	Progressive supranuclear palsy	-	NCT01049399
TDZD-8 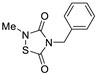	AD	Preclinical evaluation	
SB216763 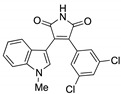	AD	Preclinical evaluation	
YQ138 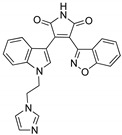	Brain ischemia	Preclinical evaluation	
Obacunone 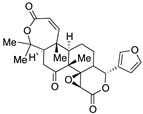	Natural product positioned toward several diseases	Preclinical evaluation	

## Abbreviations

Aβ: β-amyloid; AD: Alzheimer’s disease; AHR: aryl hydrocarbon receptor, AIP: AHR-interacting protein; ALS: amyotrophic lateral sclerosis; AMPK: AMP-activated protein kinase; ARE: antioxidant response element; ARNT: aryl hydrocarbon receptor nuclear translocator; α-syn: α-synuclein; BBB: blood–brain barrier; bHLH/PAS: basic helix–loop–helix-PER-ARNT-SIM; BTB/POZ: Bric-a-brac, tramtrac, broad-complex/proxvirus zinc fingers; β-TrCP: β-transducing repeat-containing protein Bzip: basic-region leucine zipper; CBP: [CREB (cAMP-response-element-binding protein)-binding protein]; CDK: cyclin dependent kinase; CK2: Casein kinase II; CNC: cap ‘‘n’’ collar; CTR: C-terminal domain; Cul3: Cullin 3; DGR: Dougle glycine repeat; DMF: dimethyl fumarate; EGCG: Epigallocatechin gallate; ERK: extracellular signal-regulated protein kinases; FRDA: Friedrich’s ataxia; GCL: glutamate cysteine ligase; GCLc: glutamate cysteine ligase; GI: gastro intestinal; GR: glutathione reductase; GR: glutathione reductase; GSH; glutathione; GSK-3β: Glycogen synthase kinase-3β; HD: Huntington’s disease, HNE: 4-hydroxy-2-nonenal; HO-1: heme oxygenase 1; Hrd1: ERAD-associated E3 ubiquitin ligase; HSP90: heat shock protein 90; Htt: huntingtin, IBs: ubiquitinated inclusion bodies; IκB: inhibitor of nuclear factor kappa B; IL-1β: interleukin 1β; IKK: IκB kinase; iNOS: inducible nitric oxide synthase; IVR: intervening region; JDP2: jun dimerization protein 2; JUNK: c-jun N-terminal kinases; KEAP1: Kelch-like ECH-associated protein 1; MiRNAs: MicroRNAs; MMF: monomethyl fumarate; MS: multiple sclerosis; NDDs: neurodegenerative diseases; NF-κB: Nuclear Factor-κB; NO_2_-Fas: Nitro fatty acids; NQO1: NAD(P)H/quinone oxidoreductase 1; NOX: NADPH oxidase; NRF2: nuclear factor (erythroid-derived 2)–like 2; NTR: N-terminal region; PD: Parkinson’s disease; PHLPP1/2: PH domain and leucine rich repeat protein phosphatases 1 and 2; PI3K: phosphatidylinositol-3-kinase; PIP3: phosphatidylinositol-(3,4,5)-trisphosphate; PKC: protein kinase C; PP2A: protein phosphatase; ProTa: prothymosin a; PrPSc: scrapie prion protein; PTEN: phosphatidylinositol 3,4,5-trisphosphate-3-phosphatase; ROS: Reactive oxygen species; RXRα: retinoic acid receptor-alpha; SAHA: suberolhydroxamic acid; SCF: Skp1-Cul1-F-box, SMaf: small masculoaponeurotic fibrosarcoma, SOD: superoxide dismutase, tBHQ: tert-butylhydroquinone; TDP-43: TAR DNA binding protein 43, TLRs: Toll-like receptors; TMT: trimethyltin; TNFα: tumor necrosis facor α; TSA: trichostatin A; UTR: 3′-untranslated region; VDCC: voltage dependent-calcium channels, XRE: xenobiotic response element.
